# Genome‐Wide Analysis of Protein Binding to DNA in Plastids by ptChIP‐seq

**DOI:** 10.1002/cpz1.70490

**Published:** 2026-10-31

**Authors:** V. Miguel Palomar, J. Jair López‐Morales, Sho Fujii, Andrzej T. Wierzbicki

**Affiliations:** ^1^ Departamento de Bioquímica, Facultad de Química Universidad Nacional Autónoma de México Ciudad de México México; ^2^ Graduate School of Agriculture and Life Science Hirosaki University Hirosaki Aomori Japan; ^3^ Department of Molecular, Cellular, and Developmental Biology University of Michigan Ann Arbor MI USA

**Keywords:** chloroplast transcription, chromatin immunoprecipitation, nucleoid associated proteins, plastid nucleoid

## Abstract

Plastids contain their own genomes encoding essential components of the photosynthetic apparatus and gene expression machinery. Transcription in plastids is mediated by two RNA polymerases, nuclear‐encoded (NEP) and plastid‐encoded RNA polymerase (PEP). Transcription, together with the binding of nuclear‐encoded plastid nucleoid‐associated proteins and dynamic tethering of nucleoids to plastid membranes, shapes nucleoid organization and function. Understanding these processes is critical for deciphering the regulation of gene expression in these organelles, which underpins chloroplast biogenesis, photosynthesis, and plant development. Here we present an optimized procedure for genome‐wide detection of protein‐binding sites in vivo using plastid chromatin immunoprecipitation coupled with high‐throughput sequencing (ptChIP‐seq). Compared with standard ChIP‐seq protocols, this method employs a high formaldehyde concentration for cross‐linking that maximizes DNA recovery without detectable nonspecific binding when proper controls are used. We use a custom Illumina‐compatible library preparation incorporating multiplexing strategies that enables pooling of many libraries per experiment, reducing per‐sample costs. Detailed procedures are provided for *Arabidopsis thaliana* at two developmental stages, but the method can be adapted to other species with appropriate modifications to chloroplast enrichment. Although we focus on detection of PEP, these protocols are applicable to detection of other endogenous and epitope‐tagged plastid proteins that directly interact with DNA. Unlike approaches relying on chloroplast ChIP‐qPCR or low‐resolution ChIP‐chip, ptChIP‐seq generates cost‐effective high‐resolution genome‐wide maps of binding sites of any DNA‐associated protein of interest in the plastid. This enables comprehensive investigation of active DNA metabolic processes within plastids, including transcription, replication, and DNA repair, and opens up avenues for exploring the dynamic interplay between nucleoid architecture, membrane association, and gene expression in response to developmental and environmental cues. © 2026 The Author(s). *Current Protocols* published by Wiley Periodicals LLC.

**Basic Protocol 1**: Plant material collection, chloroplast enrichment, and cross‐linking

**Alternate Protocol 1**: Working with young seedlings

**Basic Protocol 2**: Immunoprecipitation and reverse cross‐linking

**Basic Protocol 3**: DNA isolation, ptChIP validation, library preparation, and sequencing

**Basic Protocol 4**: Data analysis

## INTRODUCTION

A billion years of co‐evolution with their host cells have shaped the structure and function of the plastid genome (plastome) (Timmis et al., 2004; Wicke et al., 2011). In land plants, plastomes contain more than 100 genes encoded in 120‐ to 160‐kb chromosomes that are thought to mostly acquire a circular topology (Bendich, 2004; Bock, 2007; Herrmann et al., 1975; Kolodner & Tewari, 1975). Plastomes contain genes encoding proteins involved in photosynthesis and in gene expression machinery (Shimada & Sugiura, 1991), with some of them being transcribed as operons, reflecting the cyanobacterial ancestry of plastids (Wicke et al., 2011). The plastome is packed in a structure known as the nucleoid, which is thought to acquire a unique three‐dimensional arrangement driven by transcription and its binding to the thylakoid membrane (Hansmann et al., 1985; Krupinska et al., 2013; Lindbeck et al., 1987; Majeran et al., 2012; Palomar et al., 2024; Powikrowska et al., 2014).

Nucleoid structure in chloroplasts is likely modulated by nucleoid‐associated protein (NAP) binding. These nuclear‐encoded proteins do not have homologies with the typical NAPs found in bacteria, and their roles in supporting transcription and the nucleoid organization remain mostly unknown (Arsova et al., 2010; Garcia et al., 2008; Gilkerson et al., 2012; Grübler et al., 2017; Liebers et al., 2020; Myouga et al., 2008; Pfalz et al., 2006; Powikrowska et al., 2014; Xiong et al., 2022; Yagi et al., 2012; Yu et al., 2018).

Gene expression of plastid genes is also unique. In plants, plastome transcription involves two types of RNA polymerases: nuclear‐encoded RNA polymerase (NEP) and plastid‐encoded RNA polymerase (PEP) (Pfannschmidt et al., 2015). NEP, a single‐subunit enzyme similar to T3/T7 phage RNA polymerases, is encoded in the nuclear genome and imported into plastids. It operates primarily during the initial phases of chloroplast development (Börner et al., 2015; Weihe et al., 2012). Conversely, PEP, a multi‐subunit protein complex resembling bacterial RNA polymerases, is responsible for most transcription in mature chloroplasts. The core PEP enzyme consists of two α‐subunits (RpoA), a catalytic β‐subunit (RpoB), a β′‐subunit (RpoC1), and a β′′‐subunit (RpoC2) (Do Prado et al., 2024; Pfannschmidt et al., 2015; Pfannschmidt & Link, 1994; Ruedas et al., 2022; Vergara‐Cruces et al., 2024; Wang et al., 2024b; Wu et al., 2024). Additionally, PEP interacts with nuclear‐encoded sigma factors, essential for promoter recognition and sequence‐specific initiation of transcription (Chi et al., 2015; Puthiyaveetil et al., 2021; Wicke et al., 2011; Yagi & Shiina, 2014). In chloroplasts, PEP exists within a larger protein complex containing several supplementary subunits known as PEP‐associated proteins (PAPs), which also play vital roles in PEP activity (Ahrens et al., 2025; Do Prado et al., 2024; Steiner et al., 2011; Vergara‐Cruces et al., 2024; Wu et al., 2024).

Even though proteins encoded by plastid genes are among the most abundant on earth, our comprehension of the fundamental mechanisms governing plastid gene expression remains limited. An important tool to study chloroplast gene expression is the ability to detect protein‐DNA interactions in vivo. This may be achieved using chromatin immunoprecipitation (ChIP), which relies on the preservation of protein‐DNA interactions through chemical cross‐linking followed by pulldown using an antibody specific toward the protein of interest (Collas, 2010; Park, 2009). ChIP has been extensively used to study eukaryotic nuclear and prokaryotic genomes (Chen et al., 2018; Furey, 2012; Galagan et al., 2012; Jaini et al., 2014; Myers et al., 2015; Schmidt et al., 2009). It has also been applied to research of chloroplasts (Ding et al., 2019; Finster et al., 2013; Hanaoka et al., 2012; Kupsch et al., 2012; Newell & Gray, 2010; Noordally et al., 2013; Palomar et al., 2024, 2022; Pfalz et al., 2006; Prikryl et al., 2008; Steiner et al., 2011; Xiong et al., 2020; Yagi et al., 2012; Zhang et al., 2018; Zhong et al., 2013).

We recently introduced an optimized ChIP protocol coupled with high‐throughput sequencing to investigate protein‐DNA binding in the chloroplast genome, which we refer to as ptChIP‐seq (Palomar et al., 2022). This method addresses several challenges of studying protein‐DNA interactions in chloroplasts, including chloroplast isolation, sample quantification, protein‐DNA cross‐linking, design of appropriate negative controls, and data normalization. It also leverages unique properties of the small chloroplast genome with a custom library prep, which allows multiplexing of libraries from more than 100 samples in one lane of sequencing, leading to a very low cost per sample.

Among the main challenges in successful detection of protein‐DNA interactions by ChIP is protein‐DNA cross‐linking. We have shown that formaldehyde concentrations between 0.5% and 1%, typically used for studies of the nuclear genome (Hoffman et al., 2015; Rowley et al., 2013), are too low to successfully capture these interactions in chloroplasts (Palomar et al., 2022). Higher formaldehyde concentrations have been used to study yeast (De Jonge et al., 2020), and we use 4% formaldehyde to cross‐link chloroplasts with no evidence of nonspecific cross‐linking (Palomar et al., 2022). Cross‐linking may be performed entirely in vivo when working with 4‐day‐old seedlings. When working with older plants, cross‐linking should be done with purified chloroplasts (Palomar et al., 2022; Wang et al., 2022).

Another challenge in ChIP is finding a proper negative control (Bonhoure et al., 2014; Haring et al., 2007). When using epitope‐tagged proteins, a wild‐type control lacks the epitope and serves as a good negative control (Palomar et al., 2022). However, when using antibodies specific toward an essential protein, typically only input and no‐antibody controls are available. Such controls are acceptable as long as antibody specificity in ChIP can be verified using an independent approach (Palomar et al., 2022).

The ptChIP‐seq method may be used to detect PEP binding to the plastome when using an epitope‐tagged PAP or an antibody specific towards a core PEP subunit, RpoB (Palomar et al., 2022; Wang et al., 2024a). This method makes it possible to study genome‐wide patterns of PEP binding to DNA with substantially greater resolution and sensitivity than previously applied ChIP‐qPCR or ChIP‐on‐chip assays (Ding et al., 2019; Finster et al., 2013; Fujii et al., 2015; Hanaoka et al., 2012; Palomar et al., 2024; Prikryl et al., 2008; Wang et al., 2024a, 2022, 2021; Xiong et al., 2022; Yagi et al., 2012; Zhong et al., 2013). Moreover, PEP occupancy on individual genes may be interpreted as a proxy for transcription (Pfannschmidt et al., 1999). Considering the extensive post‐transcriptional processing in chloroplasts (Barkan, 2009; Barkan & Small, 2014; Marquardt et al., 2023; Stern et al., 2010), RpoB ptChIP‐seq is much more informative about the levels of transcription than RNA‐seq (Lee et al., 2021; Palomar et al., 2022; Xiong et al., 2022) and serves as a complementary approach to run‐on transcription (Deng et al., 1987; Isono et al., 1997; Krause et al., 2000; Krupinska & Apel, 1989; Legen et al., 2002; Melonek et al., 2010; Shiina et al., 1998; Tsunoyama et al., 2004; Valkov et al., 2009; Zubo et al., 2011) or RNA immunoprecipitation (Bobik et al., 2017; Fu et al., 2022; Hua et al., 2024; Lee et al., 2021; Lenzen et al., 2020; Melonek et al., 2010; Meurer et al., 2017; Muino et al., 2024; Schmid et al., 2019).

Our ptChIP‐seq protocol is universally applicable for testing the binding of any DNA‐binding protein, so it can be applied to other contexts beside chloroplast transcription. Unlike a previously published protocol (Wang et al., 2022), it is compatible with high‐throughput sequencing and includes a library‐generation step. In this article, we provide a detailed protocol for conducting ptChIP‐seq on both young and mature leaf tissue in *Arabidopsis thaliana*, where changes in RNA polymerase binding can be easily monitored (Palomar et al., 2024), although this technique can be applied to chloroplasts of other species.

Basic Protocol 1 covers material collection, chloroplast enrichment, and protein DNA cross‐linking before immunoprecipitation; this protocol is flexible and could be adapted depending on the organism or conditions tested. Details for working with two developmental stages of *Arabidopsis thaliana* plants are provided in Basic Protocol 1 and the accompanying Alternate Protocol 1. Basic Protocol 2 describes a general method for protein DNA immunoprecipitation and reverse cross‐linking to release DNA from the protein of interest; details about the proper use of a negative control as well as preparation for DNA recovery are provided. Basic Protocol 3 spans the processing of the DNA sample from its purification to the validation of ptChIP, qPCR validation, and library preparation for sequencing. Basic Protocol 4 provides a template for basic data analyses of sequenced data; with the fast evolution of programming and software sophistication, this protocol can be adapted for the user needs, but details are provided for non‐expert users.

*CAUTION*: Be careful when collecting tissue—the use of sharp razor blades in close contact with the skin may cause serious injuries.

*CAUTION*: When working with 2‐mercaptoethanol, formaldehyde, phenol/chloroform/isoamyl alcohol, and chloroform/isoamyl alcohol, prepare solutions in the fume hood and wear proper lab safety equipment.

*CAUTION*: Use proper noise isolation equipment when working with sonicator.

*CAUTION*: Extra care should be taken when heating microbiological needle to pinch a hole into the bottom of the tube to make the filter unit; serious burns or injuries can occur.

*CAUTION*: Proper equipment should be worn when working with liquid nitrogen or ultra‐cold material to avoid skin injuries.

## Basic Protocol 1 PLANT MATERIAL COLLECTION, CHLOROPLAST ENRICHMENT, AND CROSS‐LINKING

*Arabidopsis thaliana* seeds (Col‐0 ecotype) are sown directly on wet soil by sprinkling dry seeds. We typically sow ∼50 seeds per 5 × 5‐inch pot to obtain enough biomass for the procedure without overcrowding the pot. Trays are covered with a transparent lid, to keep humidity elevated, and incubated for 2‐4 days at 4°C in total darkness for seed stratification. Germination is performed on trays covered with transparent lids for 5 days at 22°C under white LED light (100 µmol m^−2^ s^−1^) under a 16 hr/8 hr day/night cycle; the cover is removed after the fifth day of germination or once small green plants (∼2 mm in diameter) are visible to lower humidity. Pots are watered every other day with distilled water, with liquid fertilizer added every other watering cycle. It is important to monitor light intensity to avoid photobleaching. Before chloroplast enrichment, plants should have a dark green color and not show signs of curling.

On the day of collection (after 2 weeks of growth), plants should have experienced between 1 and 2 hr of illumination before harvest. This short illumination period minimizes gene expression differences associated with prolonged darkness while limiting the starch accumulation that would occur during longer periods of illumination (Palomar et al., 2024; Fernandez et al., 2017). Harvesting is done by collecting leaf tissue with a sharp razor blade or scissors while avoiding soil debris (pots can be watered the previous day to avoid dust). Leaves can be rinsed three or four times with distilled water and dried thoroughly between two paper towels. Five grams of leaves yield enough chloroplasts for at least 10 ptChIP experiments.

Clean leaves are gently blended in chloroplast enrichment buffer using a prechilled blender. The homogenate is filtered through two layers of Miracloth (Millipore cat. no. 475855‐1R) to remove plant debris, and centrifuged for 5 min at 1500 × *g*, 4°C. All steps should be done in the cold to avoid heat‐caused damage. The green pellet is recovered by resuspension in fresh chloroplast enrichment buffer, and chloroplast DNA quantified by sequencing is enriched to 40‐60% concentration (a non‐enriched extract usually contains ∼2‐8% chloroplast reads). Chloroplasts are used directly for in vitro cross‐linking by adding formaldehyde to 4% final concentration. The sample is incubated for 4 hr on ice, and then the formaldehyde is diluted and quenched by the addition of glycine to 125 mM final concentration. Chloroplasts are pelleted to remove quenched formaldehyde and then sonicated.

### Materials

*Arabidopsis thaliana* plants, either wild type or mutant genotypes in the Col‐0 ecotype, grown on soil
Distilled water
Chloroplast enrichment buffer (see recipe)
2‐mercaptoethanol
80% acetone, prechilled at 4°C
37% formaldehyde solution, methanol free (J.T. Baker cat. no. 02‐002‐900 or similar)
Quenching buffer (see recipe)

Benchtop centrifuge with temperature control and swinging rotor for 50‐ml tubes (e.g., Eppendorf 5810R or similar), precooled to 4°C
New razor blades
Medium‐sized plastic or glass funnels
250‐ml Erlenmeyer flasks
Miracloth (Millipore cat. no. 475855‐1R)
Ice bucket
50‐ml conical‐bottom Falcon tubes (Corning cat. no. 352098 or similar)
Blender and blender cups for small volumes
Medium‐sized paintbrush
Standard vortex mixer
2‐, 20‐, 200‐, and 1000‐µl pipets with appropriate plastic tips
2‐ml plastic microcentrifuge tubes (e.g., Eppendorf)
Acetone‐resistant plastic or glass cuvettes
Visible‐light spectrophotometer
Standard high‐speed microcentrifuge

#### Plant material and growth conditions

1 Grow plants on soil using standard plant growth protocols until they reach 2 weeks of age.
2 Collect leaf tissue by cutting leaves off with a sharp razor blade, taking care to avoid any soil debris.
3 Wash the leaves three or four times with distilled water, and thoroughly dry them by pressing between paper towels.
  The leaves are now ready for chloroplast enrichment and in vitro cross linking.

#### Chloroplast enrichment

4 Assemble a filter unit for each prep by placing a medium‐sized funnel on a 250‐ml flask. Place two layers of Miracloth in the top of the funnel and chill the flask on ice.
5 Add 250 µl of concentrated 2‐mercaptoethanol to 250 ml of 1× chloroplast enrichment buffer and keep the buffer refrigerated. If performing more than one enrichment experiment, prepare 250 ml for each additional prep.
6 Chill on ice four disposable 50‐ml Falcon tubes for each prep, along with a small blender cup.
7 To avoid contamination from condensation water, pour a few milliliters of 1× chloroplast enrichment buffer into the blender cup, pour out, and discard. Place the rinsed and dried leaves (from step 3) and 150 ml of prepared 1× chloroplast enrichment buffer inside the cup and blend using around five pulses of 1‐2 sec each at medium speed. Pour the mix onto the top of the filter unit. After all the liquid has passed through the filter, gently squeeze the Miracloth to recover as much solution as possible. Clean the blender cup with water between preps.
  Do not use detergent to clean the blender cup between preps, as strong detergents may dissolve the chloroplast membrane.
8 Divide the solution between the four prechilled 50‐ml Falcon tubes and centrifuge 5 min at 1500 × *g*, 4°C, without braking.
9 Discard the supernatant and gently resuspend the pellets in the remaining buffer using a paintbrush. Transfer all the pellets together to a 2‐ml microcentrifuge tube to quantify the chlorophyll content; keep chloroplasts on ice.

#### Chlorophyll quantification

The following procedure is only suitable for genotypes with no defects in chlorophyll biogenesis. If the genotypes being studied are expected to have deficiencies in chloroplast development or pigment accumulation, use Alternate Protocol 1 instead. Chlorophyll content should be determined by spectrophotometric analysis following the method described by Porra et al. (1989).

10 Dilute 10 µl of the chloroplast solution in 1 ml of cold 80% acetone; mix by vortexing. Use another 1 ml of 80% acetone as blank.
11 Centrifuge for 2 min at 3000 × *g*, room temperature, and pour supernatant into 10‐mm acetone‐resistant plastic or glass spectrophotometer cuvettes.
12 Measure absorbance at 663.6 and 646.6 nm for chlorophyll *a* (Chl *a*) and chlorophyll *b* (Chl *b)*, respectively.
13 Calculate the total chlorophyll concentration (Chl *a* + *b*) using the following equation:
  (Chl *a*+*b*) = [(17.76*A_646.6_) + (7.34*A_663.6_)]*DF/1000
  where DF is the dilution factor. The result is expressed in mg/ml of chlorophyll.

#### In vitro cross‐linking

14 Transfer an amount of chloroplast solution equivalent to 100 µg chlorophyll to a 2‐ml microcentrifuge tube, and dilute to 200 µl in 1× chloroplast enrichment buffer.
15 Add formaldehyde to obtain a 4% final concentration (21.6 µl of 37% stock) and mix by pipetting. Incubate on ice in the dark for 4 hr. If a non‐cross‐linked control is used, prepare similarly but replacing the formaldehyde with water.
16 Dilute chloroplasts in 1650 µl quenching buffer to quench the cross‐linking reaction.
17 Centrifuge 5 min at 1500 × *g*, 4°C, with braking. Remove the supernatant and proceed with ptChIP (Basic Protocols 2‐4).
  Alternatively, pelleted chloroplasts can be stored up to 1 month at –70°C before you proceed further.

## Alternate Protocol 1 WORKING WITH YOUNG SEEDLINGS

Arabidopsis seeds (100 per 10‐cm‐diameter plate) are sterilized using ethanol followed by a bleach solution. Then, in the flow hood, three to five washes with sterile distilled water are performed. Seeds are sown on plates containing 0.5× Murashige and Skoog (MS) solid medium. Including sucrose in the medium is recommended when growing photosynthesis‐deficient mutants, but it can be omitted for plants with no obvious growth defects. Plated seeds should be stratified to homogenize germination by placing them at 4°C in total darkness for 2‐4 days and then transferring them to a growth chamber at 22°C under white LED light (50‐100 µmol m^−2^ s^−1^) under a 16 hr/8 hr day/night cycle. After 4 days, seedlings will show two green cotyledon leaves and can be collected for chloroplast enrichment. One hundred seedlings are enough to perform two ptChIP experiments.

In this procedure, collected seedlings are resuspended in water containing 4% formaldehyde and subjected to vacuum for 10 min. The vacuum is then quickly released to allow formaldehyde to enter tissue, and the samples are incubated on ice for 4 hr. Cross‐linking is stopped by adding glycine up to 600 mM and applying vacuum for 5 min. After the liquid is removed from the tubes, seedlings can either be frozen or be processed immediately for chloroplast enrichment. Enrichment is performed by blending seedlings in chloroplast enrichment buffer in a 1.5‐ml tube using a pellet pestle. The mixture is then transferred to a 0.5‐ml tube with a hole punched in the bottom that is placed over two layers of Miracloth. This filter unit is placed in a 1.5‐ml tube, which is centrifuged for 5 min at 1500 × *g* to filter and pellet the chloroplast fraction. The DNA in the resulting pellet contains ∼25‐50% plastid genome reads.

### Additional Materials (also see Basic Protocol 1)

0.5× MS plates (see recipe)
2 M glycine

Laminar‐flow hood
Sharp tweezers
1.5‐ml plastic microcentrifuge tubes
Vacuum pump and vacuum system
Bunsen burner
Microbiological needle
Mini pellet pestles
Micromotor for pellet pestles (optional)

#### Plant material and cross‐linking

1 Grow Arabidopsis plants on 0.5× MS plates in a plant growth chamber for 4 days, using standard procedures and observing good sterile practices.
2 On the day of collection, gently collect 50 seedlings using sharp tweezers and combine them in a 1.5‐ml microcentrifuge tube containing 100‐200 µl distilled water.
  If non‐photosynthetic plastids are expected to contribute to the signal of the protein of interest, restrict collection to cotyledons, where most cells contain photosynthetically active chloroplasts (Cackett et al., 2022).
3 To perform in vivo cross‐linking, add formaldehyde to 4%, mix by vortexing, expose tubes to vacuum for 2 min with open lids, release vacuum, and expose tubes to vacuum again for additional 8 min.
4 Release the vacuum, close tube lids, and incubate on ice for 4 hr.
5 After incubation, add 2 M glycine to reach 600 mM, mix by vortexing, open tube lids, and expose to vacuum for 1 min. Release the vacuum and then expose them to vacuum again for an additional 4 min.
6 Release vacuum, close tube lids, centrifuge for 1 min at >14,000 rpm in a standard microcentrifuge, and remove supernatant.
  The tissue is now ready for chloroplast enrichment. Alternatively, cross‐linked seedlings can be stored up to 3 months at –70°C before you proceed further.

#### Chloroplast enrichment

7 Prepare 500 µl of 1× chloroplast enrichment buffer by adding 0.5 µl of 2‐mercaptoethanol and keeping it cool.
8 Heat a needle using a Bunsen burner. Use this heated needle to very carefully poke two or three holes in the bottom of a 0.5‐ml microcentrifuge tube. Move the needle around a little to increase the size of each hole—the holes should be slightly larger than the diameter of the needle.
9 Assemble a filtration unit by cutting a piece of Miracloth to ∼2 × 1.5 inches. Fold this in half and use it to cover the outside of the 0.5‐ml tube. Place the whole unit into a 1.5‐ml microcentrifuge tube. Place tube on ice.
10 To a separate 1.5‐ml microcentrifuge tube, add 100 µl of 1× chloroplast enrichment buffer. Add the 50 seedlings to the tube. Using a mini pellet pestle, crush the material in the buffer.
11 Using a 1000‐µl pipet and a pipet tip with 3 mm of the end carefully cut off, transfer the liquid to the 0.5‐ml tube of the filtration unit. Spin down in a microcentrifuge for 5 min at 1500 × *g*, 4°C. Check to be sure that all of the liquid has passed into the flowthrough.
12 Remove filter unit and supernatant. Resuspend the pellet in 10 µl of 1× chloroplast enrichment buffer and proceed with immunoprecipitation (Basic Protocol 2).

## Basic Protocol 2 IMMUNOPRECIPITATION AND REVERSE CROSS‐LINKING

ptChIP is based on a previously published protocol for nuclear chromatin in plants (Rowley et al., 2013) optimized to chloroplast samples. Enriched chloroplast pellets are resuspended in a lysis buffer containing sodium dodecyl sulfate (SDS) and sonicated to obtain DNA fragments of 200‐300 bp (Fig. 1). Longer fragments can affect sequencing performance and data resolution, while shorter fragments may be eliminated during library preparation. Sonicated chloroplasts are centrifuged to eliminate insoluble material, and 2% of the supernatant is saved in an independent tube as an input control. The rest of the centrifuged supernatant is incubated with the appropriate antibody and magnetic beads. Beads containing the antibody, protein of interest, and anchored DNA are washed and subjected to reverse cross‐linking by heat and proteinase K treatments. Input samples are treated in parallel.

**Figure 1:**
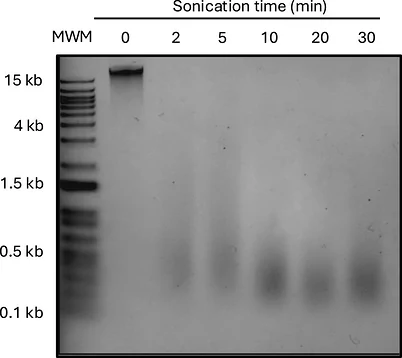
Fragments obtained after sonication of cross‐linked chloroplasts. Chloroplasts equivalent to 100 µg of chlorophyll were sonicated for the indicated time at 4°C using a water‐bath sonicator, treated with RNase A, and run in a 1% agarose gel. Molecular weight markers are shown in lane 1; selected sizes are indicated as reference.

### Materials

Cross‐linked chloroplast sample (Basic Protocol 1 or Alternate Protocol 1)
Chloroplast lysis buffer (see recipe)
Liquid nitrogen
Protein A Dynabeads (Invitrogen cat. no. 10002D)
Binding/washing (B/W) buffer (see recipe; with PMSF added just before use)
ptChIP dilution buffer (see recipe)
Antibody: anti‐RpoB (PhytoAB cat. no. PHY1239), anti‐HA (Thermo cat. no. 26183), or desired antibody for your experimental needs
TE buffer, pH 8.0 (see recipe)
ptChIP elution buffer (see recipe)
Proteinase K solution (Thermo cat. no. EO0491)

Multi‐sample water‐bath sonicator (e.g., Qsonica Q700 Cup Horn Sonicator with a Recirculating Chiller or similar), set to 4°C, and compatible tubes
Standard cooled high‐speed microcentrifuge, 4°C
2‐, 20‐, 200‐, and 1000‐µl pipets with appropriate plastic tips
1.5‐ml plastic microcentrifuge tubes
Microtube rotator (Fisher cat. no. 88861051 or similar)
Magnetic rack for microtubes (Thermo cat. no. FERMR02 or similar)
Standard vortex mixer
Heating, shaking dry bath (Thermo cat. no. 11686731 or similar)

1 Resuspend the cross‐linked chloroplasts by adding chloroplast lysis buffer to 200 µl.
2 Transfer the solution to tubes compatible with the sonicator and sonicate to obtain a fairly homogeneous population of DNA fragments of ∼200‐300 bp. Using a multi‐sample water sonicator set at 4°C, apply the following program:
  Amplitude: 22%
  Total processing time: 10 min
  Sonication time: 10 sec
  Rest time: 20 sec
  Power output: keep between 80 and 100
  Overall sonication time: 30 min
  The sonicator should be set to 4°C and takes a while to cool down, so we suggest turning it on at least 30 min ahead of time.
  The power output is determined by the amount of water in the cup; adding more water to the cup will affect the power, and therefore the power output changes when the samples are added to the cup.
  Avoid using a microtip sonicator because it tends to generate foam, given the small volumes used.
3 Centrifuge the sonicated chloroplasts in a microcentrifuge for 10 min at maximum speed, 4°C. Identify the pellet position and carefully recover the supernatant while avoiding touching the pellet.
  Pipetting the solution multiple times can result in a lot of bubbles. If this happens, after transferring the supernatant to a fresh tube, microcentrifuge it for 2 min at maximum speed, 4°C, to reduce bubbles.
4 Prepare two 98‐µl aliquots of the supernatant for ptChIP and two 2‐µl aliquots to serve as input samples and flash freeze all four aliquots in liquid nitrogen. Store at –80°C for further processing.
  This generates four aliquots in total for each sample. If the volume recovered in step 3 is <200 µl, smaller aliquots may be used, but keeping the ptChIP/input ratio at 50:1. Example: 90 µl ptChIP/1.8 µl input.
5 Place 60 µl of protein A Dynabeads in a fresh tube.
  Mix the Dynabeads well before use and between preps.
6 Dilute the beads in 1 ml B/W buffer and incubate with rotation for 5 min at room temperature. Capture beads in a magnetic rack until the supernatant becomes clear. Remove the supernatant. Repeat this step twice more, for a total of three washes. Resuspend the beads in 100 µl B/W buffer.
7 Thaw one ptChIP sample aliquot (from step 4), add 900 µl of ptChIP dilution buffer, and mix with the beads and the optimized amount of the desired antibody.
  For example, to detect RpoB binding to DNA, use 5 µg anti‐RpoB antibody; for an HA‐tagged PEP component, use 1 µg anti‐HA antibody.
  Be sure to add the components of this mixture in the order indicated, as exposing the antibody to the high SDS concentration in chloroplast lysis buffer may cause it to denature.
8 Incubate the sample/bead mixture with rotation overnight at 4°C.
  To make a no‐antibody or negative antibody control, assemble a ptChIP reaction but omit antibody addition, or add preimmune serum, if available.
  IMPORTANT NOTE: The immunoprecipitation conditions should be optimized when adapting the protocol to the use of different antibodies. In particular, antibody concentration, incubation time and temperature, as well as bead volume and incubation time, may require extensive optimization before you proceed with library preparation.
9 Briefly spin and capture the beads in a magnetic rack buried in ice until the solution becomes light green. Discard supernatant, add 1 ml B/W buffer, and rotate for 5 min at 4°C. Remove supernatant. Repeat this washing step twice more, for a total of three washes.
10 Wash once more with 1 ml of 1× TE buffer. Rotate at 4°C for 5 min and capture the beads. Resuspend beads in 55 µl ptChIP elution buffer. Vortex vigorously for 10 sec at maximum speed.
11 Thaw one aliquot of input sample (from step 4) and add 110 µl ptChIP elution buffer to it. Mix by vortexing.
12 Incubate beads in ptChIP elution buffer (from step 10) and input samples (from step 11) for 20 min at 65°C with shaking at 1400 rpm.
13 Remove the samples and briefly spin down beads. Capture beads using a magnetic rack until supernatant becomes clear. Recover supernatant and place in a fresh tube. Set aside and keep on ice. Add another 55 µl of ptChIP elution buffer to the remaining beads. Incubate the ptChIP and input sample for another 20 min at 65°C with shaking at 1400 rpm.
  Input samples do not contain beads and do not need to be spun down in this step. However, the input samples should be shaken at 65°C, 1400 rpm, twice, like the ptChIP samples.
14 Remove the samples and briefly spin down the beads. Capture beads again, recover supernatant, and mix with the supernatant obtained in the previous step.
15 Perform reverse cross‐linking by adding 3 µl proteinase K to all samples (input and eluate), mixing, and incubating for at least 2 hr at 65°C (or overnight at 50°C).
16 Let the tubes cool down and add 300 µl distilled water to each. Proceed with DNA isolation according to Basic Protocol 3.

## Basic Protocol 3 DNA ISOLATION, ptChIP VALIDATION, LIBRARY PREPARATION, AND SEQUENCING

Reverse cross‐linking is achieved through addition of an excess of proteinase K and heat incubation; incubation for 2 hr at 65°C is usually enough to ensure that the DNA will enter the aqueous phase during the subsequent 25:24:1 phenol/chloroform/isoamyl alcohol (PCI) DNA extraction. Reverse cross‐linking can be left to continue overnight at 50°C to prevent sample damage. After reverse cross‐linking, the sample is allowed to cool and then extracted with a conventional PCI protocol to purify DNA; because proteinase K and other contaminants should be removed for DNA purification, any silica‐column‐based DNA purification kit should work fine as an alternative to using phenol. If a DNA precipitation method is selected, we recommend the use of colored glycogen during DNA precipitation to avoid loss of the pellet during washing and sample handling. ptChIP experiments performed on young seedlings have DNA yields below the typical detection limit of fluorescence‐based quantification methods, but inputs can be easily quantified.

Before library generation, successful enrichment in ptChIP should be confirmed by qPCR. In case of ptChIP experiments detecting PEP binding to DNA, we use primers to amplify two PEP‐transcribed gene regions, and two negative controls composed of one NEP‐transcribed gene and one intergenic region (Fig. 2). For other proteins, prior knowledge about their binding sites is necessary at this step. ptChIP‐qPCR data is analyzed by calculating the percentage of input. Percent input values are highly dependent on the studied protein and used antibody.

**Figure 2:**
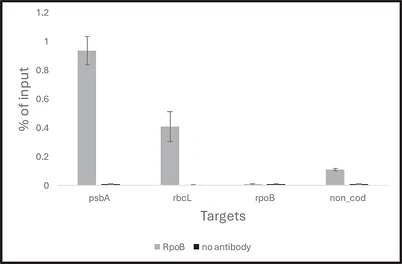
ptChIP‐qPCR of *Arabidopsis thaliana* using an antibody against the protein RpoB and compared against a no‐antibody negative control. Probes consist of two positive (PEP transcribed; *psbA* and *rbcL*) and two negative regions (not PEP transcribed; *rpoB* and a noncoding sequence representing an intergenic site between the *petA* gene and the *psbEFLJ* operon). Signal represents the average of three replicates, and error bars show standard deviation.

Library prep is performed using an Illumina‐compatible protocol (Bowman et al., 2013). Final libraries should have a size range of 200‐500 bp with a slightly stronger band leaning toward the bottom of the range (Fig. 3). Multiple dual‐barcoded libraries can be pooled together to use the same sequencing lane. Each library should be sequenced to a depth of between 1 and 2 million reads.

**Figure 3:**
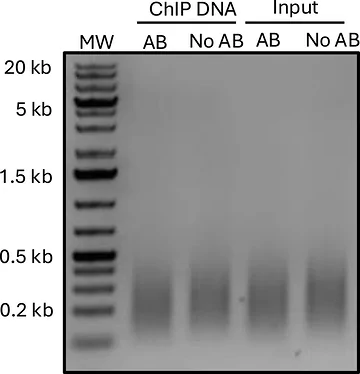
Libraries obtained from an RpoB ptChIP experiment. At least 10 ng of the libraries was run in a 1% agarose gel. Molecular weight is shown in lane 1; selected sizes are indicated as reference. Lanes 2 and 3 show libraries from ptChIP DNA, *rpoB* and no‐antibody control (No AB), respectively; and lanes 4 and 5 show input samples from *rpoB* and the no‐antibody control, respectively.

### Materials

Reverse‐cross‐linked samples (Basic Protocol 2)
25:24:1 (v/v/v) phenol/chloroform/isoamyl alcohol, pH 8.0 (Sigma cat. no. P3803)
24:1 (v/v) chloroform/isoamyl alcohol solution (Sigma cat. no. C0549)
Glycoblue (Invitrogen cat. no. AM9516)
3 M sodium acetate, pH 5.8
Isopropanol
75% ethanol supplemented with 10 mM ammonium acetate
Elution buffer (EB; see recipe)
Appropriate PCR primers for validation
iTaq Universal SYBR Green qPCR kit (Bio‐Rad cat. no. 1725120 or similar)
10× NEB End Repair Reaction Buffer (NEB cat. no. E6052A)
NEB End Repair Enzyme mix (NEB cat. no. E6051‐AA)
Ampure beads (Beckman cat. no. A63880)
80% (v/v) ethanol
10× NEB Buffer 2 (NEB cat. no. B7002S)
1 mM dATP (Roche cat. no. 11934511001)
NEB Klenow 3′‐5′ exo minus (NEB cat. no. M0212L)
10× T4 ligase Buffer (NEB cat. no. B0202S)
KAPA SYBR Green master mix (cat. no. KK4604)
1 µM universal adapter solution (Fig. 4)
T4 DNA ligase (NEB cat. no. M0202L)
p5 Illumina TruSeq barcoding primer 10 µM (Supplementary Table 1)
p7 Illumina TruSeq barcoding primer 10 µM (Supplementary Table 1)

Standard vortex mixer
Standard cooled high‐speed microcentrifuge
2‐, 20‐, 200‐, and 1000‐µl pipets with appropriate plastic tips
1.5‐ml plastic microcentrifuge tubes
Standard laboratory freezer
Real‐time PCR thermal cycler (Bio‐Rad CFX96 or similar)
PCR tubes compatible with qPCR thermal cyclers
Magnetic rack for microtubes (Thermo cat. no. FERMR02 or similar)
Heating shaking dry bath (Thermo cat. no. 11686731 or similar)
Qubit fluorometer and high‐sensitivity dsDNA quantification kit (Thermo cat. no. Q33230)

**Figure 4:**
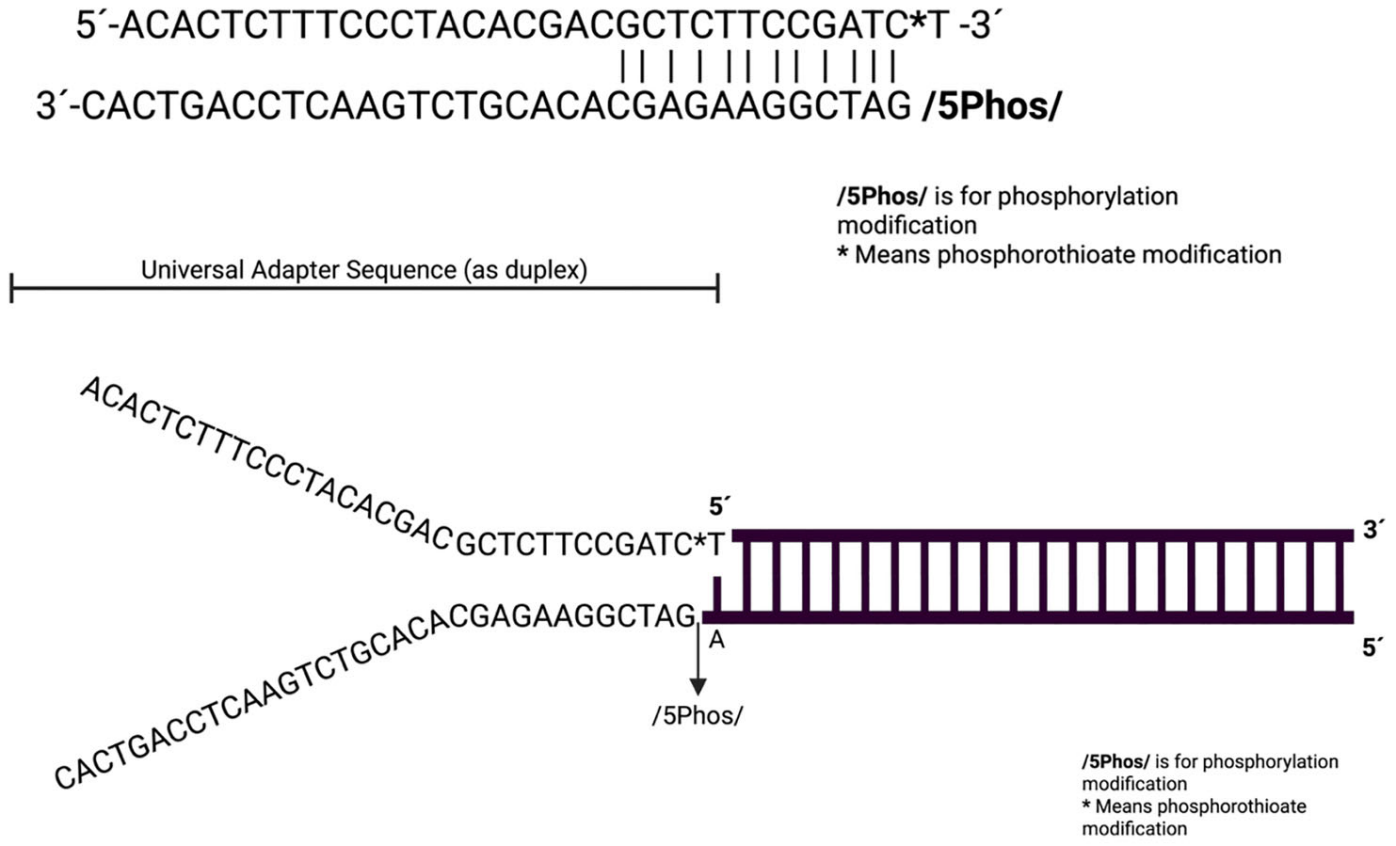
Illumina Universal Adapter sequence. Top, sequences of forward and reverse strands of the adapter; pipes are shown on complementary bases. Adapter can be ordered as duplex. Bottom, representation of one side of the fragments and its ligation with the Universal Adapter. Sticky T/A ends for ligation are shown. Figure created based on Bowman et al. (2013).

#### DNA isolation

1 Add a 1:1 volume of 25:24:1 phenol/chloroform/isoamyl alcohol to the sample (usually 400 µl). Vortex carefully for 4 sec and centrifuge for 10 min at maximum speed, room temperature.
2 Remove the upper (clear) phase, avoiding the interphase and bottom phase, and transfer to a fresh tube.
3 Add 400 µl of 24:1 chloroform/isoamyl alcohol, vortex, and centrifuge 10 min at maximum speed, room temperature.
4 Repeat step 2.
5 Add 4 µl Glycoblue, 40 µl of 3 M sodium acetate (pH 5.8), and 440 µl isopropanol. Let precipitate at −20°C for at least 2 hr (can be left overnight).
6 Centrifuge precipitated samples for 10 min at maximum speed, 4°C. Discard the supernatants.
7 Add 1 ml of 75% ethanol/10 mM ammonium acetate. Vortex and centrifuge for 5 min at maximum speed, 4°C. Discard supernatant.
8 Centrifuge for an additional 1 min at maximum speed, room temperature, and discard the remaining supernatant with a fine tip without disturbing the pellet. Air‐dry for 5 min.
9 Resuspend the pellet in 20 µl EB by pipetting.
  Optionally, RNase treatment may be performed at this point.
10 Dilute an aliquot of each sample 10× with EB and use to perform qPCR validation (steps 12 and 13).
  2 µl of DNA diluted in 18 µl EB should be enough to test up to nine or ten targets, with two technical replicates.
11 Freeze the remaining non‐diluted DNA samples to use later in performing library prep (steps 14‐21).
  DNA recovery is typically very low, so use half of the sample for the library.

#### ptChIP‐qPCR validation

Validation by qPCR can be performed by using any qPCR‐compatible reagents or even using a standard *Taq* polymerase with the addition of 1× commercial SYBR Green. We use iTaq Universal SYBR Green ready‐to‐use mix from Bio‐Rad that comes at 2× concentration. Amplification is performed on a Bio‐Rad CFX96 system, but any qPCR instrument with SYBR Green read should work fine.

12 Assemble a 10‐µl qPCR reaction mix (following the instructions of the qPCR kit or your standard procedure), and run qPCR using selected settings and PCR conditions for the chosen primers. We usually choose four primer pairs: two positive (spanning known binding sites for RpoB or the protein of interest) and two negative (Supplementary Table 1).
  For negative targets, CT values can appear after 30‐32 cycles; if this occurs, you can add five additional cycles to provide a better chance to detect a clear amplification. Positive targets usually show amplification between 22 and 26 cycles.
13 For qPCR analysis, we take replicative average Ct values for input and adjust them for the dilution factor. We express the enrichment as a percentage of 2 to the power of the difference: 100*2^(adjusted input – ChIP)^. Results are expressed as percentage of input.

#### Library preparation

For library preparation, use the undiluted ChIP DNA samples.

14 Set up a 30‐µl end‐repair reaction as follows:
  10 µl sample DNA
  15.5 µl water
  3 µl of 10× NEB End Repair reaction Buffer (NEB cat. no. E6052A)
  1.5 µl of NEB End Repair Enzyme mix (NEB cat. no. E6051‐AA)
  Incubate 30 min at room temperature.
15 Clean up the reaction with 2:1 ratio of Ampure beads to reaction mix to get rid of enzyme and buffer components:
  a. Mix Ampure beads and the DNA sample (step 14) by pipetting and incubate 5 min.
    All steps are performed at room temperature.
  b. Place tubes in magnetic rack. Wait 2 min or until the solution becomes clear, and remove supernatant.
  c. Add 200 µl of freshly prepared 80% ethanol solution and rotate the tubes 180° in the rack so that the beads move toward the opposite tube wall.
  d. Remove ethanol and repeat step c.
  e. Completely remove the ethanol and air‐dry the beads for 5 min in the rack.
  f. Remove the tubes from the rack. Resuspend the beads in 20 µl EB, vortex, and incubate 2 min.
  g. Replace the tubes in the magnetic rack and incubate until the solution becomes clear. Transfer 19 µl of the supernatant to a fresh 1.5‐ml microcentrifuge tube.
16 Set up a 30‐µl A‐tailing reaction:
  19 µl of sample DNA from the end‐repair reaction
  3 µl of 10× NEB Buffer 2 (NEB cat. no. B7002S)
  7 µl of 1 mM dATP
  1 µl NEB Klenow 3′‐5′ exo minus (NEB cat. no. M0212L)
  Incubate 30 min at 37°C.
  Clean up the reaction as in step 15 to get rid of the enzyme, buffer components, and dATP, and elute in 25 µl EB.
17 Set up a 30‐µl adapter ligation reaction:
  24 µl of sample DNA from the A‐tailing reaction
  3 µl of 10× T4 ligase buffer
  1 µl of 1 µM universal adapter (Fig. 4)
  2 µl T4 DNA ligase.
  Make sure to thaw the adapter tube on ice or at room temperature (never in your hands)
  Incubate 1 hr at room temperature (this can go longer, but 1 hr is usually enough). Clean up the reaction as in step 15 to get rid of enzyme, buffer components, and unligated adapters, and then elute in 23 µl EB. Transfer 22 µl of the supernatant to a PCR tube.
18 Set up the qPCR reaction for library amplification (50 µl):
  22 µl of sample DNA from the adapter ligation
  25 µl of KAPA SYBR Green master mix
  1.5 µl of p5 Illumina TruSeq barcoding primer (Supplementary Table 1)
  1.5 µl of p7 Illumina TruSeq barcoding primer (Supplementary Table 1)
  You can substitute KAPA SYBR Green with NEB Ultra II Q5 polymerase (NEB cat. no. M0544) with SYBR GThe freen supplementation.
19 Take 15 µl of the sample from above and run it in the qPCR machine using the following parameters (keep the rest of the sample on ice):

**Table:** 

Initial cycle:	30 sec	98°C	(denaturation)
20 cycles:	10 sec	98°C	(denaturation)
	20 sec	64°C	(annealing)
	45 sec	72°C	(elongation).
  A final extension is not needed, nor is a melting curve.
  Determine the optimum number of cycles needed to amplify the actual library from the qPCR plot (we usually use the number of cycles corresponding to 2 cycles before the plateau is reached). The qPCR reaction may be analyzed by agarose electrophoresis as an overamplification control.
20 Run a final point PCR with the remaining 35 µl of reaction that was left on ice in step 19, using the same reaction conditions but with the optimum number of cycles.
21 Purify the final library with the Ampure beads as in step 15, but using a 1.5:1 bead/sample ratio to get rid of enzyme, buffer components, and unused primers. Elute in 25 µl EB, quantify by Qubit or another fluorescence‐based method, and analyze by gel electrophoresis.
  A smear from 150 to 500 bp should be seen. If additional bands are obtained (indicating overamplification), the ratio of beads to sample may be reduced to 1:1, or gel selection may be performed. The library can be reliably analyzed and quantified using a bioanalyzer or LabChip.

## Basic Protocol 4 DATA ANALYSIS

Instructions in this paper are just for reference; as software, bioinformatics and sequencing platforms are quickly evolving, we recommend testing and building your own pipeline.

Obtained reads are adapter and quality trimmed using the Trim_galore! package (Krueger et al., 2021). Fastqc is used just to confirm good quality and sizes of reads, but in interpretating its results, take into consideration the small size, skewed GC content, and presence of inverted repeats in the chloroplast genome. Processed reads are usually mapped against the latest chloroplast genome (TAIR12 as of the date of this article) using bowtie2 (Langmead & Salzberg, 2012) with standard options, mapping rate should show chloroplast enrichment between 40 and 60%. The rest of the reads usually map to the nuclear genome with a very small fraction mapped to mitochondria. Mapped files are further processed to build coverage files where reads are annotated for each nucleotide on the genome for visualization in the open source plastid‐specific genome browser Plavisto https://plavisto.mcdb.lsa.umich.edu/ (Palomar et al., 2022; Fig. 5).

**Figure 5:**
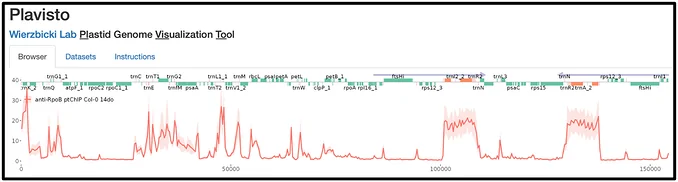
Screenshot of the Plavisto tool displaying signal obtained from ptChIP for the protein RpoB. The upper section shows the gene and regional organization of the *Arabidopsis thaliana* chloroplast genome. The red line represents the average signal from different biological replicates in the selected dataset; the ribbon shows the standard deviation calculated for each nucleotide or the selected number of bins.

### Materials

Reads obtained from sequencing core or company
Computing equipment with at least 8 cores, 32 Gb RAM memory, and 2 Tb internal or cloud storage
Trim_galore! package (Krueger et al., 2021)
SAMtools
BEDTools

1 After sequencing, download your data to the destination disk. Typically, fastq files are gzip compressed.
2 Adapter trim and quality filter reads using Trim_galore!:


trim_galore --paired --fastqc Filename_1.fastq.gz Filename_2.fastq.gz

  Use the --paired option if the sequencing mode was paired‐end. Use --fastqc to perform a quality check.
  Replace filenames with your own file names for each sample, or use an automated naming method.
3 Build an index with your reference genome (if all of your samples will be mapped to the same reference, this index needs to be generated only once to process them):


bowtie2‐build /path/to/your/genome/genome.fasta index_base

  Provide genome file in fasta format.
  Use an index_base to identify your genome reference (e.g., Arabidopsis_tair_10).
4 Map your reads to the genome reference:


bowtie2 ‐x /path/to/your/genome/index_base ‐q ‐N 1 ‐p 10 --no‐mixed --no‐discordant --fr ‐1 Filename_1_val_1.fq.gz ‐2 Filename_2_val_2.fq.gz ‐S Your_sample.sam

  Use the ‐x option to select your genome reference.
  The ‐N option allows 1 error per seed region.
  The number of processor cores can be changed by modifying the numeric value under the ‐p option.
  The --no‐mixed, --no‐discordant, and --fr options are used to keep biologically logical paired reads.
  Use ‐1 and ‐2 to indicate your processed files from Trim_galore!
  The ‐S option saves the file in .sam format.
5 Process your mapped file with SAMtools to build the coverage‐per‐nucleotide files:


samtools view ‐bf 0 × 2 Your_sample.sam > Your_sample.bam
samtools sort ‐n Your_sample.bam ‐o Your_sample_sorted.bam
samtools fixmate Your_sample_sorted.bam Your_sample_fixed.bam

  Use samtools view to convert your .sam file to a binary format .bam file using only proper paired reads (‐bf 0 × 2 options).
  Sort your reads by name using samtools sort ‐n.
  Ensure paired‐end read information is correct with samtools fixmate.
6 Generate .bed files with paired information:


bamToBed ‐bedpe ‐mate1 ‐i Your_sample_fixed.bam | awk '{OFS="\t"}{if($9=="+") print"$1,$2,$6,$7,$8,$9"; else print "$1,$5,$3,$7,$8,$9"}' | awk '{if($2>0) print $0}' | sortBed ‐i stdin > Your_sample.bed

  Use the options ‐bedpe and ‐mate1 to set bedpe format and to report read 1 information first.
Awk processing is used to format reconstructed fragments so that the start coordinate will be the same as that of read 1 and the end coordinate the last coordinates of read 2. If reads are mapped on the passenger strand, coordinates will be inverted, and if negative coordinates are obtained, they will be eliminated.
  The file is sorted for coverage file generation using sortBed.
7 Optionally, generate coverage‐per‐nucleotide maps to display your samples using Plavisto (https://plavisto.mcdb.lsa.umich.edu/).
  a. Create a file with your genome sizes (needs to be done only once):

echo ‐e “Pt\t0\t154477” > Chloroplast_genome_size.txt

  Your file will contain the chromosome name “Pt” and start and end coordinates (0 and 154477 for Arabidopsis thaliana).
  The ‐e option allows echo to use regular expressions and include tabs as separator “\t”.
  b. Generate the coverage files:

coverageBed ‐d ‐a Chloroplast_genome_size.txt ‐b Your_sample.bed >
Your_sample_coverage.bed

  Use the option ‐d to generate coverage‐per‐nucleotide files.
  Options ‐a and ‐b are used to select your genome coordinates and sample, respectively.

## REAGENTS AND SOLUTIONS

### Binding/washing (B/W) buffer

150 mM NaCl
20 mM Tris·Cl, pH 8.0
2 mM EDTA, pH 8.0
1% (v/v) Triton X‐100
0.5% (v/v) SDS

Add 10 µl of 1 mM phenylmethylsulfonyl fluoride (PMSF; diluted in ethanol) per 1 ml of B/W buffer right before use. The final solution should contain 10 µM PMSF.

A small stock of B/W buffer with PMSF added can be made right before use; leftover buffer should be discarded after 1 day. Buffer without PMSF may be stored up to 6 months at 4°C and should be mixed well before every use.

### Chloroplast enrichment buffer

0.33 M sorbitol
30 mM HEPES

Calibrate pH to 7.5 with KOH, filter through 0.22‐µm‐pore‐size nylon membrane, and keep cold. Solution can be prepared and stored as a 5× stock at 4°C, if desired. Buffer may last up to 6 months under good sterile conditions.

### Chloroplast lysis buffer

50 mM Tris·Cl, pH 8.0
10 mM EDTA, pH 8.0
1% (w/v) SDS
Store up to 6 months at 4°C
  Solution should be warmed before use because SDS tends to precipitate in cold temperatures.

### Elution buffer (EB)

10 mM Tris·Cl, pH 7.5‐8.0
Store up to 6 months at 4°C

### MS plates, 0.5×

1.25 g/liter Murashige and Skoog (MS) salt mixture
0.5 g/liter 2‐(*N*‐morpholino)ethanesulfonic acid (MES)‐KOH, pH 5.7
0.65% (w/v) agar

Autoclave mixture and carefully pour into 100‐mm round petri dishes. Dishes can be stored up to 1 month at 4°C.

### ptChIP dilution buffer

1.1% Triton X‐100 (v/v)
1.2 mM EDTA, pH 8.0
16.7 mM Tris·Cl, pH 8.0
167 mM NaCl
Store up to 6 months at 4°C

### ptChIP elution buffer

100 mM Tris·Cl, pH 8.0
10 mM EDTA, pH 8.0
1% (w/v) SDS
Store up to 6 months at 4°C
  Solution should be warmed before use because SDS tends to precipitate in cold temperatures.

### Quenching buffer

1× chloroplast enrichment buffer (see recipe)
125 mM glycine
Store up to 2 months at 4°C under good sterile conditions

### TE buffer

10 mM Tris·Cl, pH 8.0
1 mM EDTA, pH 8.0
Store up to 6 months at 4°C

## COMMENTARY

### Background Information

The procedure presented here addresses several longstanding challenges in the study of protein‐DNA interactions in the chloroplast and other plastids. The use of 4% formaldehyde cross‐linking maximizes DNA yield and allows the use of minimal amount of tissue. This is especially important when working with plants with obvious developmental defects, when getting enough tissue for genomic analysis is complicated. The comparison of two developmental stages in this article is important as the binding of certain proteins changes depending on plastid differentiation.

Depending on what protein is being screened by ptChIP‐seq, an appropriate negative control should be chosen. This procedure is flexible in regard to the use of an untagged line as well as a no‐antibody control, essential when working with proteins for which a viable knockout mutant cannot be obtained. Custom library preparation is another advantage of ptChIP‐seq. Because the chloroplast genome is small, current sequencing technologies give higher‐than‐needed yields, allowing several hundred samples to be multiplexed at the same time in a cost‐effective manner. This allows the production of more biological replicates within the same experiment or the addition of different negative controls to increase certainty about an observed pattern. Although the ptChIP‐seq procedure presented here focuses on detection of the plastid transcription machinery, the protocols can be adapted to any protein for which an antibody is available, such as nucleoid‐associated proteins, replication machinery proteins, promoter recognition sigma factors, or any other directly DNA‐bound protein. Overall, ptChIP‐seq represents a powerful addition to the plant molecular biology toolkit, enabling systematic investigation of the mechanisms governing plastid gene expression, which is central to plant growth, development, and metabolism.

### Critical Parameters

#### Selection of the right tissue for ptChIP

To obtain good and reliable results, researcher should select a genotype and growth conditions in which the protein of interest shows good accumulation and the expected DNA‐binding activity. These protocols have been tested mainly for soft tissues (leaves and seedlings) where the most chloroplasts are expected; working with harder tissues will introduce additional challenges for tissue processing. The protocols have been adapted for the use of small samples; if a researcher expects a low abundance of the protein of interest, we recommend increasing the amount of tissue used to increase the chances of good immunoprecipitation.

#### Antibody validation

ptChIP‐seq depends on good antibody detection of the target protein, so the antibody should be validated before use in this protocol; we recommend confirming by western blot that a clear and specific detection occurs. In our experience, even not‐that‐clean results on a western blot do not indicate that the antibody cannot be used in these protocols, but in case antibody detection is not 100% specific, negative controls become more important. We therefore recommend adding as many negative controls as needed (non‐tagged lines, mutant genotypes, preimmune serum, and/or no‐antibody controls).

#### Use of validated DNA binding regions

After immunoprecipitation, it is important to know whether detection was specific and thus whether it is safe to proceed with sequencing libraries. In this case, for qPCR validation, the researcher should select validated DNA regions where the protein of interest should be bound. In case of RpoB, we designed primers against two protein‐coding genes that were previously demonstrated to be transcribed by the PEP complex, and selected one region not transcribed by PEP and one intergenic region as negative targets. In our experience, a good immunodetection should show at least a 10‐fold more enrichment of a positive target compared to a negative control, but this can depend on many factors, such as protein abundance and binding dynamics. For proteins without known binding regions but that are known to contain DNA‐binding domains, we perform ptChIP by adding as many negative controls as possible and with at least five biological replicates. Even when we must skip qPCR validation, we can interpret results after sequencing as long as negative controls show cleaner and different (mostly random) enrichment patterns between replicates, while the ptChIP‐seq samples show a reproducible pattern.

#### Selection of the right primer pair for multiplex experiments

One advantage of this method is that it allows one to multiplex sequencing libraries and reduce the cost per sample in sequencing experiments. The researcher should be sure that libraries are planned before selecting the barcoding primers that will be used for each sample. Ten p5 and ten p7 barcoding primers will produce 100 libraries, so careful planning should be performed before adding each primer to each library prep. After sequencing, samples will be identified by the combination of p5 and p7 barcodes, and there is no other way to trace back a sample if the barcoding primers were mixed during library prep.

### Troubleshooting

Common problems with the protocols, their causes, and potential solutions are described in Table 1.

**Table 1:** Troubleshooting Guide for ptChIP‐qPCR, Library Prep, and ptChIP‐seq

Problem	Possible cause	Solution
Similar enrichment of positive and negative targets	Immunodetection failed or cross‐linking was inefficient	Verify specificity by western blot. When verified, modify bead and antibody concentrations used. Make sure that your formaldehyde solution is not expired and your vacuum source works correctly.
Obtained enrichment was >100% or <0.001% of input	DNA recovery on input or ptChIP samples was very low	Be sure to mix Glycoblue well before every use. Ensure that you are not aspirating the DNA pellet during extraction.
No cq values obtained in qPCR	Faulty polymerase amplification	Make sure that the isolated DNA was eluted in EB and not in ptChIP elution buffer (the latter contains SDS and EDTA and will inhibit qPCR). If EB buffer was correctly used, check that your qPCR kit is working by amplifying other targets in an unrelated DNA sample.
No amplification in library prep qPCR	Residual ethanol in library cleaning steps or faulty polymerase amplification	Make sure that after each Ampure bead cleaning step, ethanol completely evaporates. Test your qPCR kit by amplifying an unrelated sample.
Smear in gel of amplified library has fragments >500 bp	Deficient chloroplast sonication	Sonicate a chloroplast sample, isolate DNA, treat with RNase A, and run in a gel; if the smear is out of range, modify sonication time, water tank volume and/or sonication intensity.
No consistent signal between replicates after sequencing	Failed immunodetection or mixed p5 and p7 primers	Make sure qPCR validation is consistent between replicates before library prep. Make a plan to assign p5 and p7 primers with a unique combination between libraries.
A high peak signal on an individual nucleotide is visible after sequencing	Low sequencing coverage	If a single high peak after sequencing is not expected, make sure to assign at least 1 million mapped reads to each sample; because chloroplast mapping is ∼40‐50% after enrichment, it is best to assign at least 2 million raw reads per library.

### Understanding Results

After qPCR validation, the comparison between positive and negative targets must show significant and clear difference in enrichment (Fig. 2); this difference is required to validate that the immunoprecipitation worked correctly and it is safe to proceed to library preparation. Sometimes, depending on the protein of interest, DNA binding might be weak or transient and the dynamic range between selected positive and negative targets might be low. In this case, if sequencing is limited, we advise verifying other regions in the genome where the protein should be bound, and that might show higher enrichment, by qPCR and comparing the results to those for negative regions. If the binding pattern of the protein is completely unknown, the researcher might use a chloroplast region as positive binding control and nuclear or mitochondrial regions as negative binding controls, as long as the protein tested is known to specifically accumulate in the chloroplast. The last resort would be sequencing without qPCR validation, but this option is risky, as it requires incurring the effort and cost of sequencing without knowing if the ptChIP experiment was successful.

After library preparation, DNA quantification by Qubit usually gives a figure in the range of 0.5‐10 ng/µl in our hands; a lower concentration may be hard to sequence, and higher concentrations may carry a risk of overamplification and artifactual enrichment of non‐random regions after sequencing. Running 10 ng of final library on a 1% agarose DNA gel should result in a smear between 200 and 500 bp (Fig. 3); we advise using light dye buffers in the gel so that they won't interfere with smear signals (an orange dye is best for this purpose). The protocol allows the pooling of large numbers of samples (>100), but the feasibility of pooling depends on the sequencing technology used. The researcher should be sure to send enough library DNA for successful sequencing and keep enough backup library and original ptChIP DNA to use in case the sample needs to be re‐sequenced or regenerated.

After sequencing and data analysis, high‐resolution maps of protein‐binding sites can be obtained without much processing. Comparing ptChIP samples and input is essential for assessing sequencing bias due to the difference in DNA base content between regions in the chloroplast genome (Fig. 5). Because the chloroplast genome is small, an additional signal normalization might be required; for that purpose, we advise using previous knowledge of the binding behavior of the protein of interest or a spike in control.

### Time Considerations

Basic Protocol 1—Collection of plant material, chloroplast enrichment, and cross‐linking: 8 hr

Alternate Protocol 1—Working with young seedlings: 8 hr

Basic Protocol 2—Immunoprecipitation and reverse cross‐linking: 20 hr

Basic Protocol 3—DNA isolation, ptChIP validation, library preparation, and sequencing: 16 hr

Basic Protocol 4—Data analysis: timing depends on researcher approach

### Author Contributions

**V. Miguel Palomar**: Conceptualization; funding acquisition; investigation; methodology; supervision; writing—original draft; writing—review and editing. **J. Jair López‐Morales**: Investigation. **Sho Fujii**: Conceptualization; investigation; methodology. **Andrzej T. Wierzbicki**: Conceptualization; funding acquisition; supervision; writing—review and editing.

### Conflict of Interest

Authors declare no conflict of interest

## Supporting information




Supplementary Table 1. Primer sequences used in this protocol

## Data Availability

Any requests for data contained in this protocol should be addressed to the corresponding authors.
